# Potential of NRF2 Inhibitors—Retinoic Acid, K67, and ML-385—In Overcoming Doxorubicin Resistance in Promyelocytic Leukemia Cells

**DOI:** 10.3390/ijms251910257

**Published:** 2024-09-24

**Authors:** Michał Juszczak, Paulina Tokarz, Katarzyna Woźniak

**Affiliations:** Department of Molecular Genetics, Faculty of Biology and Environmental Protection, University of Lodz, Pomorska 141/143, 90-236 Lodz, Poland; paulina.tokarz@biol.uni.lodz.pl (P.T.); katarzyna.wozniak@biol.uni.lodz.pl (K.W.)

**Keywords:** apoptosis, doxorubicin, leukemia, NRF2, reactive oxygen species, resistance

## Abstract

Drug resistance is one of the major obstacles to the clinical use of doxorubicin, an extensively used chemotherapeutic drug to treat various cancers, including leukemia. Inhibition of the nuclear factor erythroid 2-related factor 2 (NRF2) seems a promising strategy to reverse chemoresistance in cancer cells. NRF2 is a transcription factor that regulates both antioxidant defense and drug detoxification mechanisms. In this study, we investigated the potential of three inhibitors of NRF2—K67, retinoic acid and ML-385—to overcome doxorubicin resistance in promyelocytic leukemia HL-60 cells. For this purpose, low-dose doxorubicin was used to establish doxorubicin-resistant HL-60/DR cells. The expression of NRF2 and its main repressor, Kelch-like ECH-associated protein 1 (KEAP1), at mRNA and protein levels was examined. HL-60/DR cells overexpressed NRF2 at mRNA and protein levels and down-regulated KEAP1 protein compared to drug-sensitive HL-60 cells. The effects of NRF2 inhibitors on doxorubicin-resistant HL-60/DR cell viability, apoptosis, and intracellular reactive oxygen species (ROS) levels were analyzed. We observed that NRF2 inhibitors significantly sensitized doxorubicin-resistant HL-60/DR cells to doxorubicin, which was associated with increased intracellular ROS levels and the expression of *CAS-9*, suggesting the participation of the mitochondrial-dependent apoptosis pathway. Furthermore, ML-385 inhibitor was used to study the expression of NRF2–KEAP1 pathway genes. NRF2 gene and protein expression remained unchanged; however, we noted the down-regulation of KEAP1 protein upon ML-385 treatment. Additionally, the expression of NRF2-regulated antioxidant and detoxification genes including *SOD2*, *HMOX2*, and *GSS* was maintained upon ML-385 treatment. In conclusion, our results demonstrated that all the studied inhibitors, namely K67, retinoic acid, and ML-385, increased the efficacy of doxorubicin in doxorubicin-resistant HL-60/DR cells, and suggested a potential strategy of combination therapy using NRF2 inhibitors and doxorubicin in overcoming doxorubicin resistance in leukemia.

## 1. Introduction

The nuclear factor erythroid 2-related factor 2 (NRF2) is a transcription factor that plays a major role in maintaining cellular redox homeostasis and in protecting cells from oxidative stress [1,2,3]. The expression of NRF2-regulated genes is induced by the binding of NRF2 to the antioxidant response element (ARE) in the regulatory regions. NRF2-regulated genes include stress response genes such as the heme oxygenase-1 (*HMOX1*) gene, antioxidant genes encoding superoxide dismutase 2 (*SOD2*), glutathione peroxidase 2 (*GPX2*), glutathione synthetase (*GSS*), glutathione S-transferase 3 (*GST3*) and drug-metabolizing enzymes, and drug transporters genes such as the NAD(P)H dehydrogenase (quinone) 1 (*NQO1*) gene.

NRF2 is tightly regulated, mainly by its repressor, Kelch-like ECH-associated protein 1 (KEAP1), an adaptor subunit of Cullin 3-based ubiquitin E3 ligase complex (Cul3) [4]. Under homeostatic conditions, NRF2 is maintained at low levels in the cytosol through its interaction with KEAP1, which directs NRF2 toward proteasome. This constitutive degradation of NRF2 ensures only the basal expression of NRF2 target genes. However, under oxidative stress, sensor cysteines of KEAP1 are oxidized, allowing NRF2 to dissociate from KEAP1 and thus escape KEAP1-mediated proteasomal degradation [5]. Liberated NRF2 translocates into the nucleus and binds to the ARE elements, and then activates transcription of the target genes. The regulation of NRF2 is not restricted to post-transcriptional KEAP1-dependent regulation but includes both post-transcriptional KEAP1-independent regulation and transcriptional regulation.

Although the NRF2–KEAP1 signaling pathway plays a cytoprotective role in normal cells and tissues, its aberrant activation has been demonstrated in many types of tumors such as lung, breast, head and neck, ovarian, and endometrial cancers [4]. NRF2 high activity was associated with increased cancer progression and resistance to anticancer treatment, and thus was considered a poor prognostic factor [6]. Moreover, in vitro studies showed that the overexpression of NRF2 increased during acquisition of drug resistance [7,8]. Increased survival and drug resistance was associated with high activation of NRF2 and was attributed to the elevated expression of target genes such as antioxidant enzymes, detoxifying enzymes, and drug efflux transporters [9,10,11], whereas NRF2 down-regulation sensitized cancer cells to chemotherapeutic agents [10]. Collectively, these results demonstrate that high activity of NRF2 can play a role in chemoresistance observed in many cancer types, and thus, the inhibition of NRF2-dependent antioxidative response seems an attractive approach to render cancer cells more susceptible to chemotherapeutic agents.

Doxorubicin, a member of the anthracycline family, is among the most widely used chemotherapeutic drugs for treating cancer, including breast, ovary, bladder, lung and thyroid cancers, sarcomas, and blood cancers including acute lymphoblastic leukemia, acute myeloblastic leukemia, and Hodgkin lymphoma [12,13]. The anticancer properties of doxorubicin are mainly exerted by its intercalation with DNA, inhibition of DNA topoisomerase II activity, and increased generation of reactive oxygen species (ROS) [14,15]. Clinical application of doxorubicin is currently limited by the occurrence of dose-dependent cardiotoxicity and doxorubicin resistance [16]. Recent studies showed that doxorubicin resistance is associated with the generation of free radicals, including ROS and activation of NRF2. Since the primary role of NRF2 signaling is the protection of cells against oxidative stress, it is assumed that targeting NRF2 can have beneficial effects in overcoming cancer cell resistance to doxorubicin [17].

**Figure 1:**
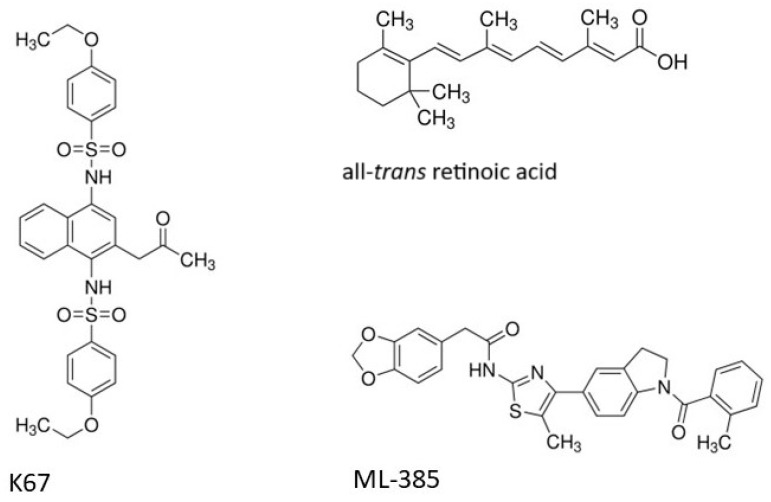
Chemical structures of NRF2 inhibitors.

In this study, we investigated the possibility of overcoming doxorubicin resistance in HL-60 cells by three NRF2 inhibitors: all-*trans*-retinoic acid (RA), K67, and ML-385 (Figure 1). RA inhibited NRF2 transcriptional activity via a mechanism involving RARα (retinoic acid receptor α) activation [18,19,20]. RA is clinically used to treat acute promyelocytic leukemia (APL) and also has been studied as a preventative and therapeutic agent against several other types of cancer. RA blocks NRF2 by activating the RARα–NRF2 complex and causes ROS accumulation, leading to ROS-dependent cytotoxicity [21]. Treatment with decitabine (DAC) alone resulted in the induction of ROS and the activation of the NRF2–ARE antioxidant response. Co-treatment with RA diminished the binding of HDAC1 (histone deacetylases 1) to the RARα–NRF2 complex, thereby preventing the activation of NRF2 by DAC alone and consequently blocking the transcriptional activation of NRF2 downstream antioxidant genes [21].

Quantitative high-throughput screening (qHTS) of 155,000 compounds allowed for the identification of K67 as an inhibitor of complex formation between phosphorylated protein p62 (*p*-p62) and KEAP1 [22]. The interaction of *p*-p62 with KEAP1 led to the constitutive activation of NRF2 which resulted in chemoresistance of cancer cells [23]. K67 inhibited NRF2 by disturbing the formation of the *p*-p62–KEAP1 complex. Currently, work on new derivatives of K67 is in progress. These derivatives potentially increase the sensitivity of cancer cells to anticancer drugs such as sorafenib or regorafenib [24]. ML-385 is a compound discovered by qHTS out of approximately 400,000 compounds of the Molecular Libraries Small Molecule Repository (MLSMR) [25]. ML-385 has been shown to directly interact with NRF2 by binding to the Neh1 domain, which allows for NRF2 interaction with DNA, especially with ARE regions [26]. ML-385 inhibits interaction of NRF2 with sMAF (small musculoaponeurotic fibrosarcoma transcription factors) and down-regulates transcription of NRF2-dependent genes [27]. Additionally, ML-385 antagonizes the NRF2MAFG (Transcription Factor G) transcriptional complex by disrupting the interaction between the CNC-bZIP (Cap’n’Collar - Basic Leucin Zipper) domain of NRF2 and MAFG, thereby inhibiting their binding to DNA regulatory sequences [25]. Moreover, ML-385 inhibits the PI3K–mTOR (Phosphoinositide 3-kinase—Mammalian Target of Rapamycin) signaling pathway in lung squamous cell carcinoma [28].

We hypothesized that NRF2 inhibition could result in reduced antioxidant response, which could overcome doxorubicin resistance. For this purpose, we chose relatively low concentrations of doxorubicin to observe the modulation of doxorubicin-induced cytotoxicity. The concentrations of tested NRF2 inhibitors were selected according to the literature [18,22,23]. To verify this, we generated a doxorubicin-resistant HL-60 cell line (HL-60/DR) and observed an increased level of NRF2 at mRNA and protein levels and a decreased level of KEAP1 protein without a change in mRNA level. As expected, HL-60/DR were more resistant to doxorubicin as evaluated by cell viability, apoptosis, and intracellular ROS induction assays. Subsequently, we demonstrated that all NRF2 inhibitors sensitized HL-60/DR to doxorubicin through decreased cell viability, increased apoptosis, and elevated intracellular ROS level, especially in the case of ML-385. Finally, we studied the molecular mechanism of ML-385-induced reversal of doxorubicin resistance. We analyzed the expression of NRF2 and KEAP1 at mRNA and protein levels as well as the expression of NRF2-dependent genes such as *SOD2*, *HMOX2*, *GSS*, and three caspases: executive *CAS-3*, extrinsic pathway *CAS-8*, and intrinsic pathway *CAS-9*.

## 2. Results

### 2.1. Comparison of HL-60 and HL-60/DR Cells

**Figure 2:**
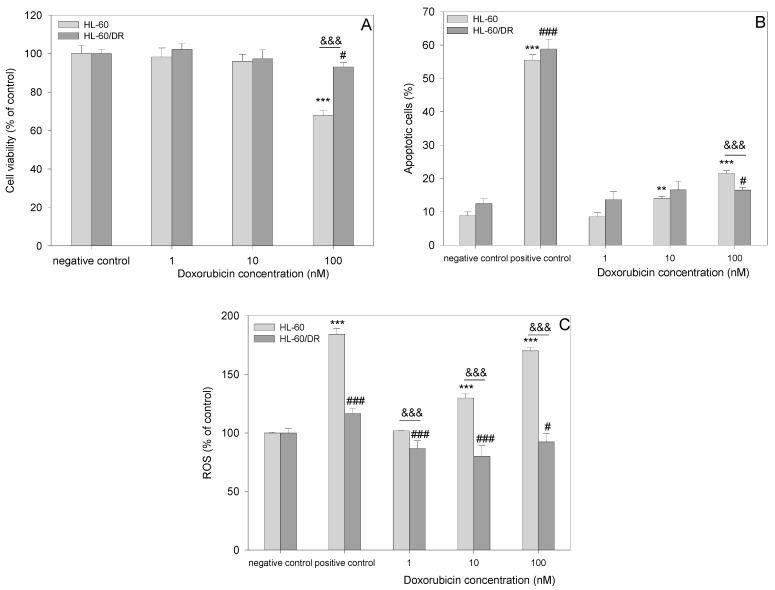
Effects of doxorubicin in HL-60 and HL-60/DR cells. (**A**) Cell viability, (**B**) apoptosis, and (**C**) intracellular ROS level. The data are presented as mean ± SD, *n* = 6. Statistical analysis was conducted using the U Mann–Whitney test, ** *p* < 0.01, *** *p* < 0.001 compared to HL-60 cells negative control, ^#^ *p* < 0.05, ^###^ *p* < 0.001 compared to HL-60/DR cells negative control, and ^&&&^ *p* < 0.001 HL-60 cells compared to HL-60/DR cells at the same conditions.

Firstly, we performed experiments that confirmed differences between HL-60 and HL-60/DR cells in response to doxorubicin, including cell viability, apoptosis, and intracellular ROS level (Figure 2). We observed differences in cell viability after incubation with doxorubicin between HL-60 and HL-60/DR cells, especially at the concentration of 100 nM (*p* < 0.001) (Figure 2A). Doxorubicin at 100 nM significantly reduced the viability of HL-60 cells (viability at 68%) (*p* < 0.001) and slightly reduced the viability of HL-60/DR cells (viability at 94%) (*p* < 0.05) (Figure 2A). Doxorubicin at 1 and 10 nM did not affect cell viability in both types of cells. Next, we demonstrated differences in apoptosis level after incubation with doxorubicin between HL-60 and HL-60/DR cells (Figure 2B). Doxorubicin at 100 nM significantly induced apoptosis in HL-60 cells (*p* < 0.001) and slightly in HL-60/DR cells (*p* < 0.05). The level of apoptosis in doxorubicin-sensitive HL-60 cells was significantly higher than in HL-60/DR-resistant cells (*p* < 0.001). Doxorubicin at 1 and 10 nM did not induce apoptosis in both types of cells. Finally, we performed the analysis of the intracellular ROS level in HL-60 and HL-60/DR cells in response to doxorubicin (Figure 2C). Doxorubicin significantly increased ROS level at the concentrations of 10 and 100 nM in HL-60 cells (*p* < 0.001) and was significantly higher than in HL-60/DR cells (*p* < 0.001). In the case of HL-60/DR cells, we observed a significant decrease in ROS level at all concentrations of doxorubicin. The obtained result confirmed that HL-60/DR effectively reduced oxidative stress in response to doxorubicin when compared to native HL-60 cells.

**Figure 3:**
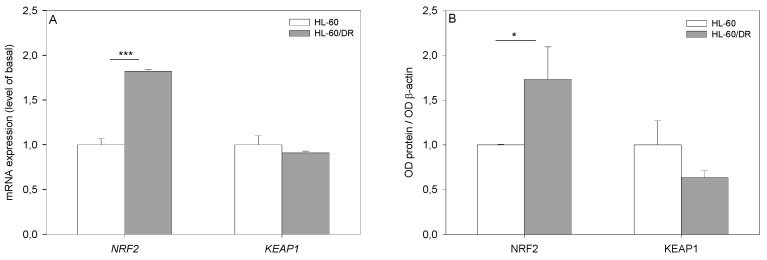
Relative expression of NRF2 and KEAP1 at mRNA (**A**) and protein (**B**) levels in HL-60 and HL-60/DR cells. Gene expression was analyzed by TaqMan real-time reverse transcription PCR and normalized to *GAPDH* gene expression. Protein expression of NRF2 (#12721, Cell Signaling Technology, Danvers, MA, USA) and KEAP1 (#8047, Cell Signaling Technology, Danvers, MA, USA) were visualized by Western blot. The intensity of bands corresponding to proteins was analyzed by densitometry. Protein expression of β-actin (sc-477778, Santa Cruz Biotechnology, Santa Cruz, CA, USA) served as the loading control. The data are presented as mean ± SD, *n* = 4, * *p* < 0.05, *** *p* < 0.001. Statistical analysis was conducted using the U Mann–Whitney test.

Next, we analyzed the expression of NRF2 and its main repressor, KEAP1, in HL-60 and HL-60/DR cell lines. We observed that NRF2 is overexpressed in HL-60/DR cells at both gene (Figure 3A) and protein (Figure 3B) levels compared to HL-60 cells (*p* < 0.001 and *p* < 0.05, respectively). We also noted no changes in KEAP1 expression at gene (Figure 3A) and protein (Figure 3B) levels. In summary, we confirmed that HL-60/DR cells were more resistant to doxorubicin when compared to HL-60 cells. Moreover, we directly associated doxorubicin resistance with overexpression of NRF2. We believed that the obtained results were a prerequisite for further studies on NRF2 inhibition as an approach for abolishing doxorubicin resistance in promyelocytic leukemia cells.

### 2.2. Cell Viability Studies

**Figure 4:**
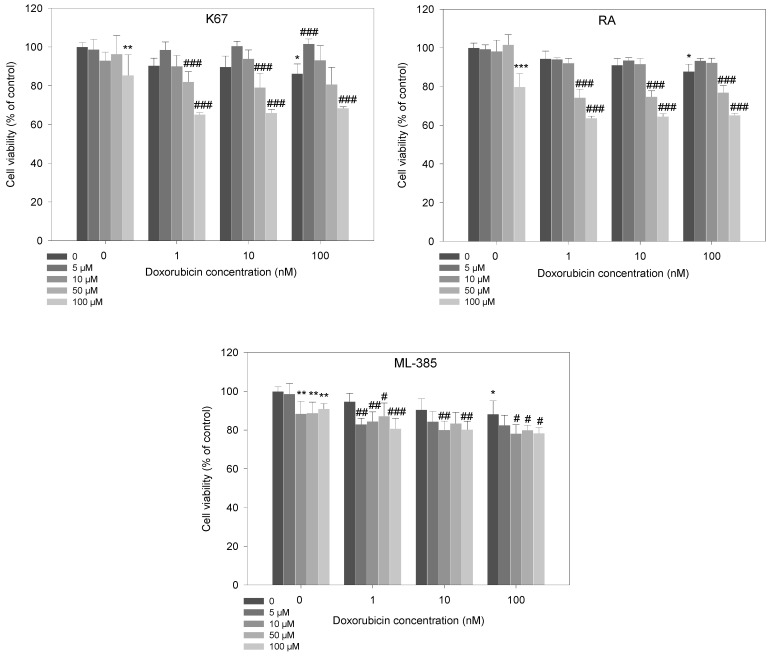
HL-60/DR cell viability pre-incubated for 24 h with NRF2 inhibitors—K67, retinoic acid (RA) and ML-385—and treated with doxorubicin for a subsequent 24 h. The data are presented as mean ± SD, *n* = 6. Statistical analysis was conducted using the U Mann–Whitney test, * *p* < 0.05, ** *p* < 0.01, *** *p* < 0.001 compared to control without doxorubicin and inhibitors, and ^#^ *p* < 0.05, ^##^ *p* < 0.01, ^###^ *p* < 0.001 compared to control with doxorubicin and without inhibitor.

Our results showed that HL-60/DR cells were resistant toward doxorubicin and the cytotoxic effect was observed only at 100 nM doxorubicin (*p* < 0.001) (Figure 4). Next, we pre-treated HL-60/DR cells with NRF2 inhibitors and observed that all studied NRF2 inhibitors significantly reduced viability of HL-60/DR cells in response to 100 nM of doxorubicin (Figure 4). K67 and RA decreased cell viability with doxorubicin at 50 and 100 nM (*p* < 0.001), and ML-385 reduced cell viability already at the concentrations of 5 and 10 nM (*p* < 0.01).

### 2.3. Apoptosis

**Figure 5:**
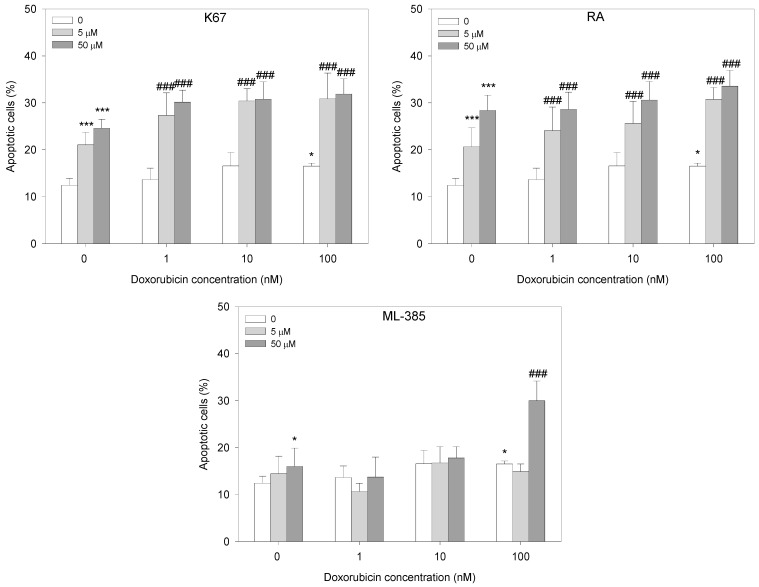
Apoptosis of HL-60/DR cells pre-incubated with NRF2 inhibitors—K67, retinoic acid (RA) and ML-385—for 24 h and treated with doxorubicin for a subsequent 24 h. The data are presented as mean ± SD, *n* = 6. Statistical analysis was conducted using the U Mann–Whitney test, * *p* < 0.05, *** *p* < 0.001 compared to control without doxorubicin and inhibitors, and ^###^ *p* < 0.001 compared to control with doxorubicin and without inhibitor.

Following the cell viability assay, we analyzed the induction of apoptosis in HL-60/DR cells with and without inhibitors in response to doxorubicin. Each tested inhibitor, except for ML-385 at 5 μM, significantly increased the population of apoptotic cells. Pre-incubation of HL-60/DR cells with K67 and RA increased apoptotic cells at all doxorubicin concentrations compared to doxorubicin alone (*p* < 0.001). In the case of ML-385, we observed doxorubicin-induced apoptosis only in HL-60/DR cells pre-incubated with ML-385 at 50 µM and then incubated with doxorubicin at 100 nM (*p* < 0.001) (Figure 5).

### 2.4. Reactive Oxygen Species Generation

**Figure 6:**
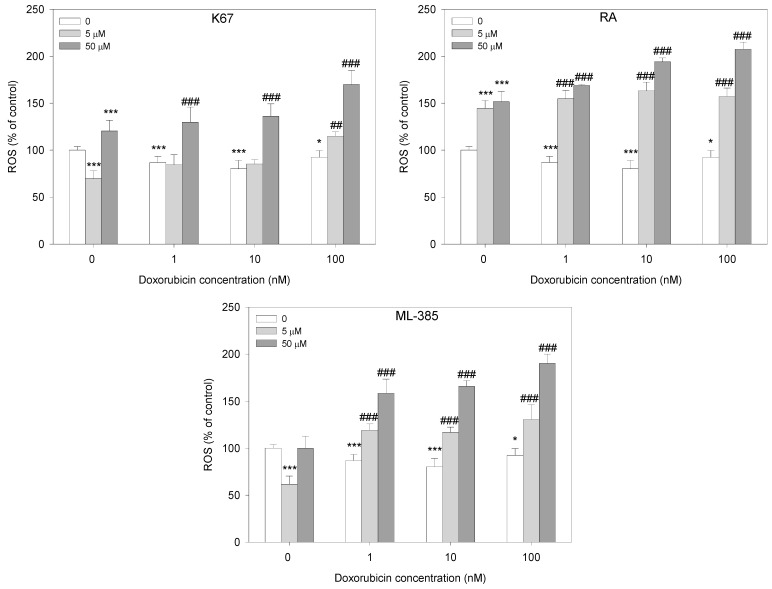
ROS level in HL-60/DR cells pre-incubated with NRF2 inhibitors—K67, retinoic acid (RA) and ML-385—for 24 h and treated with doxorubicin for a subsequent 24 h. The data are presented as mean ± SD, *n* = 6. Statistical analysis was conducted using the U Mann–Whitney test, * *p* < 0.05, *** *p* < 0.001 compared to control without doxorubicin and inhibitors, and ^##^ *p* < 0.01, ^###^ *p* < 0.001 compared to control with doxorubicin and without inhibitor.

Since oxidative stress imbalance is implicated in cancer cell resistance, we took a closer look at the intracellular ROS level in doxorubicin-treated HL-60/DR cells in combination with NRF2 inhibitors. We observed a tendency toward increased ROS level in HL-60/DR cells pre-incubated with NRF2 inhibitors compared to doxorubicin itself (Figure 6). In particular, all inhibitors at a concentration of 50 μM were effective in a whole range of doxorubicin concentrations tested (*p* < 0.001). Moreover, cells increased ROS in a similar manner when RA and ML-385 were used at a concentration of 5 μM in response to doxorubicin (*p* < 0.001).

Doxorubicin itself did not increase ROS level in HL-60/DR cells. On the contrary, the drug decreased endogenous ROS at all used concentrations.

### 2.5. Gene Expression Analysis

**Figure 7:**
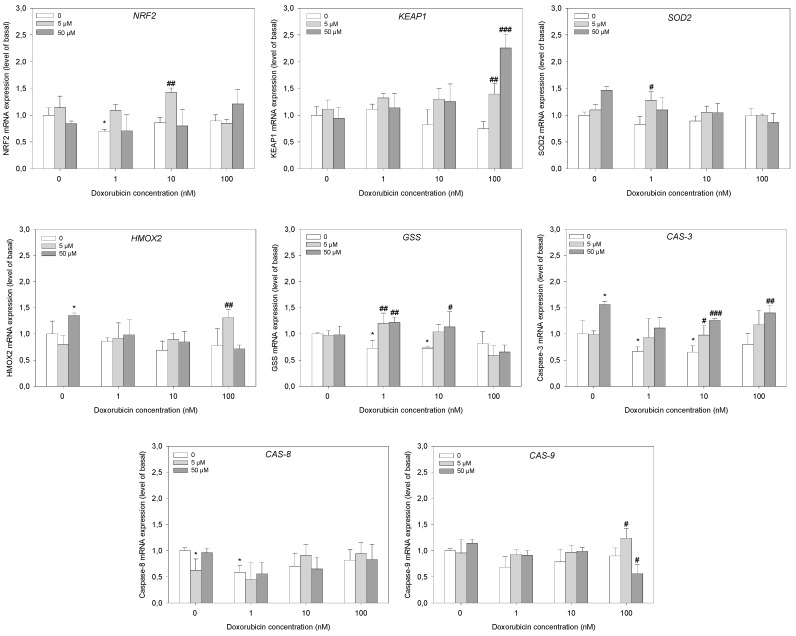
Relative expression of *NRF2*, *KEAP1*, *SOD2*, *HMOX2*, *GSS*, *CAS*-3, *CAS*-8, and *CAS*-9 genes in HL-60/DR cells pre-incubated with ML-385 for 24 h and treated with doxorubicin for a subsequent 24 h. The figures show mean results ± SD, *n* = 4, * *p* < 0.05 compared to control without doxorubicin and ML-385, and ^#^ *p* < 0.05, ^##^ *p* < 0.01, ^###^ *p* < 0.001 compared to control with doxorubicin and without ML-385. Statistical analysis was conducted using the U Mann–Whitney test.

For further gene expression experiments in HL-60/DR cells, we chose ML-385. ML-385 reduced cell viability similarly to RA and K67 but when applied alone, it did not induce significant apoptosis and ROS, and thus, we consider ML-385 to be the most neutral inhibitor. Otherwise, the results obtained with RA and K67 could interfere and demonstrate a synergistic effect of the compound and not the effect of NRF2 inhibition. We studied the effect of ML-385 on *NRF2* gene expression, its main repressor *KEAP1*, NRF2-dependent genes such as *SOD2*, *HMOX2*, *GSS*, and three genes of caspases *CAS-3*, *CAS-8*, and *CAS-9* (Figure 7). Neither *NRF2* nor *KEAP1* mRNA levels were modified by ML-385. The observed point increase in *NRF2* expression in the case of pre-incubation with 5 µM ML-385 and then incubation with 10 nM doxorubicin is most likely a fluctuation of qPCR and with a high degree of probability that it does not exhibit biological effects. Unexpectedly, ML-385 at all concentrations significantly increased the expression of the *KEAP1* mRNA level when HL-60/DR cells were faced with 100 nM doxorubicin. In the case of ML-385 at 50 µM, we observed 2.2-fold increase (*p* < 0.001).

In the case of *SOD2* gene expression, a statistically significant change was observed only for the combination of 5 µM ML-385 and 1 nM doxorubicin (*p* < 0.05). For the *HMOX2* gene, the mRNA level was increased for 5 µM ML-385 and 100 nM doxorubicin (*p* < 0.01). Moreover, 50 µM ML-385 slightly increased the *HMOX2* mRNA level by itself (*p* < 0.05). Doxorubicin slightly decreased the mRNA level of the *GSS* gene at 1 and 10 nM (*p* < 0.05). Pre-incubation with ML-385 caused a visible increase in the *GSS* mRNA level for 1 nM doxorubicin (*p* < 0.01). Expression of the *CAS-3* gene was decreased by 1 and 10 nM doxorubicin (*p* < 0.05). ML-385 increased the *CAS-3* mRNA level at 50 µM (*p* < 0.05). Pre-incubation of HL-60/DR cells with ML-385 at this concentration significantly increased the *CAS-3* level for doxorubicin at 10 and 100 nM (*p* < 0.001 and *p* < 0.01, respectively). We observed a slight decrease in the *CAS-8* mRNA level for 5 µM ML-385 itself (*p* < 0.05) and 1 nM doxorubicin (*p* < 0.05). In the case of the mRNA level of *CAS-9*, an increase was observed for a combination of 5 µM ML-385 and 100 nM doxorubicin (*p* < 0.05). Surprisingly, the mRNA level decreased for 50 µM ML-385 and 100 nM doxorubicin (*p* < 0.05) (Figure 7). However, it should be noted that the observed increase in expression of *NRF2* and *HMOX2* is lower or approximately 1.5-fold and could not have a biological impact on increasing expression of protein.

### 2.6. Western Blot Analysis

**Figure 8:**
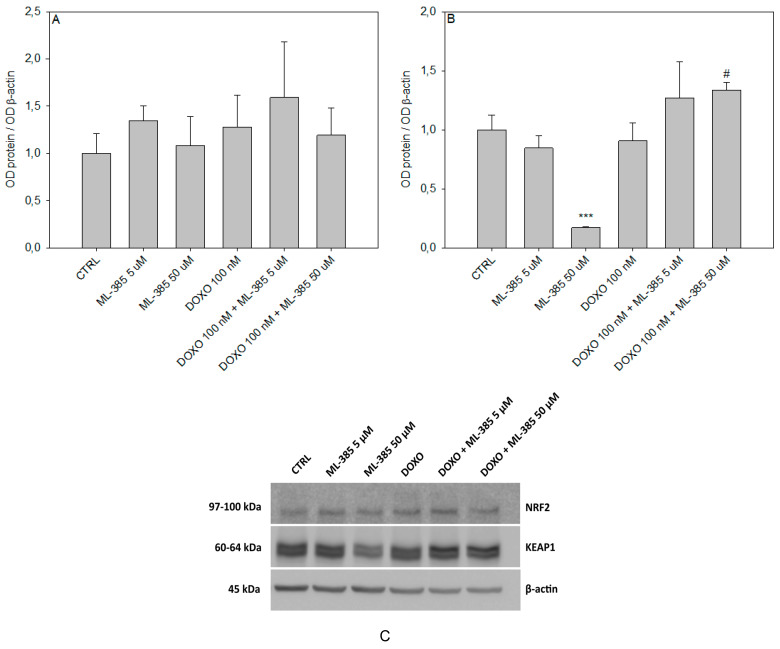
Protein expression of NRF2 (#12721, Cell Signaling Technology, Danvers, MA, USA) (**A**,**C**) and KEAP1 (#8047, Cell Signaling Technology, Danvers, MA, USA) (**B**,**C**) in HL-60/DR cells pre-incubated with ML-385 for 24 h and treated with 100 nM doxorubicin (DOXO) for a subsequent 24 h was visualized by Western blot. The intensity of bands corresponding to proteins was analyzed by densitometry. The results are shown as the fold change of proteins levels of treated cells vs. control HL-60/DR cells. β-actin (sc-477778, Santa Cruz Biotechnology, Santa Cruz, CA, USA) served as the loading control. The figure shows mean results ± SD, *n* = 4, *** *p* < 0.001 compared to control without doxorubicin and ML-385, and ^#^ *p* < 0.05 compared to control with doxorubicin and without ML-385. Statistical analysis was conducted using the U Mann–Whitney test.

In addition to the gene expression assay, we analyzed the expression of NRF2 and KEAP1 on the protein level by Western blot (Figure 8). We have observed that neither ML-385 nor doxorubicin treatment had an influence on NRF2 protein level in HL-60/DR cells. However, increasing concentration of ML-385 systematically reduced the level of KEAP1. Moreover, we observed that HL-60/DR cells pre-treated with ML-385 and faced with doxorubicin increased the level of KEAP1 protein (*p* < 0.05), which corresponds to the observed elevation of *KEAP1* transcription (Figure 7).

## 3. Discussion

In this study, we have addressed the impact of NRF2 inhibition on overcoming doxorubicin resistance in HL-60/DR cells. Doxorubicin resistance acquired during chemotherapy is the major obstacle to the successful treatment of many cancers. Recently, it was demonstrated that clinical doxorubicin-resistant leukemia samples significantly overexpressed NRF2 [29]. Similarly, we have observed that doxorubicin-resistant HL-60/DR demonstrated overexpression of NRF2 at mRNA and protein levels. Since the parental HL-60 cell line was doxorubicin-sensitive, we assumed that acquired resistance in HL-60/DR cells may be associated with NRF2 overexpression. The activity of NRF2 is tightly controlled by its repressor, KEAP1. Therefore, we investigated the modulation of KEAP1 in HL-60/DR cells and found that KEAP1 protein level was down-regulated when compared to drug-sensitive HL-60 cells. To the best of our knowledge, HL-60/DR cells do not bear *KEAP1* mutation [30] but increase NRF2 expression while gaining doxorubicin resistance. It thus seems that exclusively targeting NRF2 can be beneficial in chemoresistant cancer cells.

Overcoming doxorubicin resistance clinically requires both the study of the molecular mechanism of cancer cells resistance to doxorubicin and the exploration of new strategies to reverse doxorubicin resistance. That is why much attention has been directed toward targeting the NRF2–KEAP1 pathway to break chemoresistance. We investigated three NRF2 inhibitors, namely K67, RA, and ML-385. We observed that each NRF2 inhibitor sensitized doxorubicin-resistant HL-60/DR cells to the drug. Several mechanisms underlying the doxorubicin resistance are considered, including generation of intracellular ROS [31,32]. We demonstrated that each NRF2 inhibitor increased the intracellular ROS level in HL-60/DR cells. Given that NRF2 inhibitors can simultaneously increase intracellular ROS and block *NRF2* transcriptional activity, our results indicate that targeting NRF2 via K67, RA, and ML-385 has the potential to increase doxorubicin-induced cytotoxicity. RA together with doxorubicin acted synergistically in the MCF-7 cell line, decreasing cell viability and increasing apoptosis [33,34]. Indeed, K67 specifically overcame sorafenib resistance in HCC cells without an impact on drug-sensitive hepatocellular carcinoma cells (HCC) after 72 h incubation [23]. Moreover, K67 suppressed the proliferation and tolerance to sorafenib and cisplatin in HCC cells [22]. Unexpectedly, K67 was effective in overcoming doxorubicin resistance in HL-60/DR already after 24 h pre-incubation in our study. ML-385 increased anticancer activity of doxorubicin in non-small cell lung cancer (NSCLC) cells with constitutive activation of NRF2 due to *KEAP1* homozygous point mutations [25]. The combination of ML-385 with glutathione peroxidase 4 (GPX4) inhibitor synergistically targeted leukemia cells triggering ferroptosis [35]. We observed that ML-385 potentiated the toxicity of doxorubicin in HL-60/DR cells. Moreover, ML-385 exerted the cytotoxic effect already after 24 h pre-incubation in HL-60/DR cells, rather than in NSCLC cells, which were co-incubated with ML-385 and chemotherapeutic drugs for 72 h [23]. Combined treatment with ML-385 and autophagy inhibitor increased cytotoxicity to cisplatin in CD44^+^ cancer stem cells demonstrating remarkable resistance toward cisplatin [36]. Apart from synthetic inhibitors, some natural compounds such as ailanthone [37], kaempferol [38], chaetominine [39], and triptolide [29], which down-regulate NRF2 activity and generate oxidative stress, sensitized drug-resistant cancer cells.

Recently, it was shown that ML-385 reduced the expression of *NRF2* and simultaneously induced the expression of its inhibitor *KEAP1* in NSCLC cells with *KEAP1* loss-of-function mutations [40]. On the contrary, we observed that ML-385 had no effect on NRF2 protein expression in doxorubicin-resistant HL-60/DR cells but decreased the expression of KEAP1 protein after 24 h treatment. In vivo research performed on C57BL/6 mice confirmed the ability of ML-385 to decrease KEAP1 protein expression [41]. Interestingly, upon doxorubicin treatment, ML-385 up-regulated the expression of KEAP1 protein in doxorubicin-resistant HL-60/DR cells, indicating the possibility of breaking doxorubicin resistance by overexpression of KEAP1. It should be emphasized that both NRF2 and KEAP1 have a broad interactome, indicating a complexity of possible interactions [42]. KEAP1 interactome demonstrates that this protein participates in many cellular processes especially in regulating actin cytoskeleton, proteostasis, or mitochondrial function [43]. Due to the multitude of possible factors influencing the protein expression of NRF2 and KEAP1, it is difficult to clearly indicate the mechanism of KEAP1 overexpression after pre-incubation with ML-385 in response to doxorubicin compared to ML-385 alone. A similar trend was observed in lung cancer cell line A549, where an ROS inductor, hydrogen peroxide, decreased protein expression of KEAP1. However, pre-incubation of A549 with ML-385 and then incubation with hydrogen peroxide significantly increased KEAP1 expression compared to hydrogen peroxide alone [44].

Moreover, we took a closer look at the expression of NRF2-regulated genes upon using NRF2 inhibitors. SOD2 catalyzes the dismutation of the superoxide radical and HMOX2 is a sensor of oxidative stress. Recently, it was demonstrated that clinically doxorubicin-resistant leukemia cells overexpressed *SOD2* [38]. Taken together, it seems that the modulation of the intracellular ROS level has the potential to break resistance to doxorubicin in an NRF2-dependent manner. Additionally, we noted the increased gene expression of initiator caspases *CAS-8* and *CAS-9* and executioner caspase gene *CAS-3* in HL-60/DR cells pre-treated with ML-385 and faced with doxorubicin.

An important aspect of the potential clinical use of the NRF2 inhibitors, including ML-385, is their interactions with anticancer drugs. The combination of ML-385 with cisplatin derivatives enhanced cytotoxic effects in head and neck squamous cell carcinoma (HNSCC) cells [45], in BT16 cells [a cell line derived from childhood patients suffering from ATRT (atypical teratoid rhabdoid tumors)] [46], and in vivo in NSCLC cells [25] compared to drugs alone. Quinacrine, an antimalarial agent and NRF2 inhibitor, reversed 5-fluorouracil (5-FU) resistance in colorectal cancer (CRC) cell lines by decreasing the expression of NRF2 [47]. Luteolin, a natural inhibitor of NRF2, enhanced the efficacy of cisplatin in HCT116 and SW620 cell lines [48]. Brusatol (another natural NRF2 inhibitor) and luteolin increased the cytotoxic potential of 5-FU against gastric cancer cell line MKN-45 [49]. Thus, NRF2 inhibitors could be considered as a potential strategy to increase the cytotoxic effect of anticancer drugs, including doxorubicin, cisplatin, and 5-FU.

Some NRF2 inhibitors have already been tested using in vivo models. ML-385 inhibited proliferation of lung squamous cell carcinoma (LUSC) organoids, whereas NRF2 promoted growth of LUSC cells in vivo [28]. 

Doxorubicin is a broad-spectrum anticancer drug still extensively used in the treatment of various cancers due to its high efficacy. Unfortunately, epidemiological data indicate that long-term administration of doxorubicin can cause severe side effects, the primary side effect being cardiotoxicity [50]. Doxorubicin increased expression of KEAP1 on the protein level in cardiac cells of Sprague-Dewley rats [51]. Doxorubicin-induced oxidative stress is the major cause of damage to cardiomyocytes. Unfortunately, doxorubicin-induced cardiotoxicity is mostly related to NRF2 suppression. It is well documented that activation of NRF2 signaling protected cardiomyocytes by inhibiting ROS expression levels [52]. Thus, the in vivo studies on the combination of NRF2 inhibitors and doxorubicin are crucial. ML-385 decreased the cardioprotective effect of (1) an antioxidant, asiaticoside, in cardiomyocytes of C57BL/6 mice [53], (2) an NRF2 inductor, DL-3-*n*-butylphthalide, in rat cardiomyocytes H9C2 in vitro [54], and (3) an NRF2 indirect inductor, tranilast, in H9C2 cardiomiocytes [55].

Besides cardiotoxicity, the administration of doxorubicin in clinical settings is correlated with neurotoxicity [50]. ML-385 reduced the neuroprotective effect of antioxidants: (1) procyanidins in ICR mice [56], (2) salidroside in C57BL/6 mice with induced Parkinson’s Disease-like symptoms [57], and (3) hydrogen sulfide in rat PC12 neuronal cell line [58].

Although NRF2 inhibitors exert beneficial effects in overcoming doxorubicin resistance in vitro, their introduction into clinical practice requires careful consideration. Preclinical studies demonstrated that administration of NRF2 inhibitors in order to reverse chemoresistance could be limited by more severe side effects, including cardiocytotoxicity and neurotoxicity. Bearing in mind the broad interactome of NRF2 and KEAP1, more holistic studies on the dual role of the NRF2–KEAP1 pathway are still needed.

Although the data on NRF2 inhibitors (K67, RA, and ML-385) obtained from this study are coherent, we cannot exclude the possibility that the observed sensitization to doxorubicin can be cell-specific. We only employed an APL HL-60 cell line which lacks the expression of p53. Further studies encompassing different leukemia cell lines, including primary leukemia cells, are needed. The broad interactome of NRF2 and KEAP1 encourages us to the consider the wide-ranging analysis of protein interactions. 

## 4. Materials and Methods

### 4.1. Chemicals

ML-385, K67, RA, doxorubicin hydrochloride (doxorubicin), 2′,7′-dichlorofluorescein diacetate (H_2_DCF-DA), Hank’s balanced salt solution (HBSS), dimethyl sulfoxide (DMSO), and hydrogen peroxide (H_2_O_2_) were purchased from Sigma-Aldrich (St. Louis, MO, USA). All other chemicals were of the highest commercial grade available. Stock solutions of ML-385, K67, and RA (10 mM) were prepared in DMSO, and a stock solution of doxorubicin (1 mM) in DNase/RNase-free water. Chemical structures of used NRF2 inhibitors are shown in Figure 1.

### 4.2. Cell Culture

A HL-60 (human promyelocytic leukemia) cell line was obtained from the American Type Culture Collection (ATCC) and cultured in Iscove’s Modified Dulbecco’s Medium (IMDM) medium (Biowest, Nuaillé, France) with 15% fetal bovine serum (Biowest, Nuaillé, France) and streptomycin/penicillin solution (100 μg/mL and 100 U/mL) (Biowest, Nuaillé, France). HL-60 cells were cultured in flasks at 37 °C in 5% CO_2_ and sub-cultured every 2–3 days to maintain exponential growth.

The HL-60/DR (doxorubicin-resistant) cell line was derived from the HL-60 cell line by long-term exposure to continuous stepwise increase of doxorubicin concentration as we described previously [59].

### 4.3. Experimental Schemes

**Figure 9:**
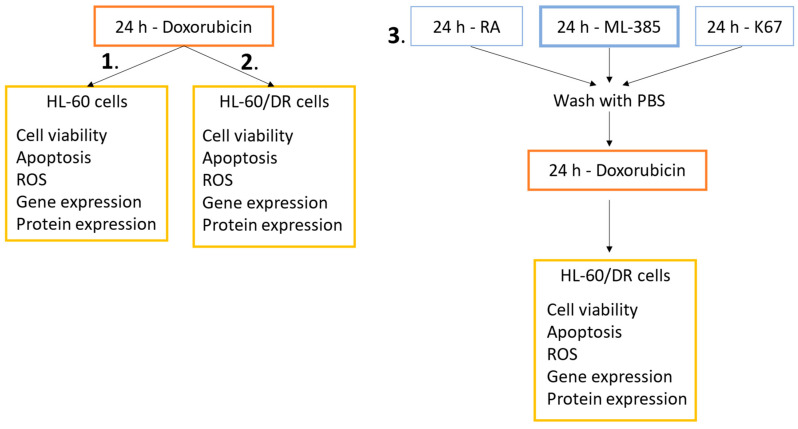
Experimental schemes of HL-60 and HL-60/DR cells. (1) HL-60 cells and (2) HL-60/DR cells were incubated with doxorubicin. (3) HL-60/DR cells were pre-incubated with NRF2 inhibitors followed by doxorubicin treatment.

Experiments were performed with three schemes of cell treatment: HL-60 and HL-60/DR cells were incubated with doxorubicin for 24 h (schemes 1 and 2, respectively); and HL-60/DR cells were pre-incubated with NRF2 inhibitors for 24 h followed by the incubation with doxorubicin for 24 h (scheme 3) (Figure 9). We investigated cell viability, apoptosis, ROS level, and gene and proteins expression in HL-60 and HL-60/DR cells.

### 4.4. Cell Viability Resazurin Assay

The cell viability resazurin assay was performed similarly to the method described by O’Brien et al. [60]. Resazurin salt powder was dissolved in sterile PBS buffer. Cells were seeded onto 96-well plates in the count of 10,000 cells per well. Doxorubicin at concentrations 1, 10, and 100 nM was added for 24 h in experimental scheme 1 and 2. In the case of experimental scheme 3, HL-60/DR cells were seeded in 25 cm^2^ flasks at a density of 2 × 10^5^ cells/mL. Then, cells were incubated with NRF2 inhibitors at concentrations of 5, 10, 50, and 100 μM for 24 h. After this time, cells were washed two times with warm PBS and seeded onto 96-well plates in the count of 10,000 cells per well. Then, doxorubicin at concentrations 1, 10, and 100 nM was added for a subsequent 24 h. Finally, 10 μL of resazurin salt was added to each well, and the plates were incubated again for 2 h. The fluorescence was measured with HT microplate reader Synergy HT (BioTek Instruments, Winooski, VT, USA) using λ_ex_ = 530/25 and λ_em_ = 590/35 nm. The results were expressed as a percentage of control cells (100%) incubated in medium only.

### 4.5. Evaluation of Oxidative Stress

In order to measure the production of intracellular ROS, the fluorescence of H_2_DCFDA was measured. H_2_DCFDA is a cell-permeable non-fluorescent probe, which is de-esterified intracellularly and turns into highly fluorescent product upon oxidation. Cells at a density of 1 × 10^6^ cells/mL were incubated with 1, 10, and 100 nM doxorubicin for 24 h in experimental scheme 1 and 2. In the case of experimental scheme 3, HL-60/DR cells were seeded in 25 cm^2^ flasks at a density of 5 × 10^5^ cells/mL. Then, the cells were incubated with NRF2 inhibitors at concentrations of 5 and 50 μM for 24 h. After this time, cells were washed two times with warm PBS and seeded onto 6-well plates in the count of 200,000 cells per well in medium. Then, doxorubicin at concentrations 1, 10, and 100 nM was added for a subsequent 24 h. Finally, cells were washed twice with HBSS containing Ca^2+^ and Mg^2+^ and stained with 20 μM H_2_DCFDA for 30 min at 37 °C in the dark. Then, cells were washed twice with HBSS and incubated at 37 °C in the dark. The intensity of fluorescence was measured with λ_ex_ = 495 nm and λ_em_ = 530 nm using a microplate reader Synergy HT (BioTek Instruments, Winooski, VT, USA). The data were analyzed according to the following formula: [(T_x_ − T_0_)/T_0_] × 100, where T_x_ is the fluorescence measured at the indicated time point and T_0_ is the fluorescence measured at the beginning of the analysis. 5 mM H_2_O_2_ as a positive control. The results were expressed as a percentage of control cells (100%) incubated in medium only.

### 4.6. Apoptosis

The FITC Annexin V Apoptosis Detection Kit II (BD Biosciences, PA, USA) was used to evaluate the induction of apoptosis using flow cytometry. Cells were seeded onto 6-well plates at a density of 2 × 10^5^ cells/mL per well. Doxorubicin at concentrations 1, 10, and 100 nM was added for 24 h in experimental scheme 1 and 2. In the case of experimental scheme 3, HL-60/DR cells were seeded in 25 cm^2^ flasks at a density of 5 × 10^5^ cells/mL. Then, cells were incubated with NRF2 inhibitors at concentrations of 5 and 50 μM for 24 h. After this time, cells were washed two times with warm PBS and seeded onto 6-well plates at a density 2 × 10^5^ of cells/mL. Then, doxorubicin at concentrations 1, 10, and 100 nM was added for a subsequent 24 h. Finally, cells were collected and washed three times with ice-cold PBS. Then, cells were resuspended in 100 µL of 1× Binding Buffer and transferred to cytometry tubes. FITC annexin V (5 µL) and propidium iodide (5 µL) were added to cells in suspension, gently vortexed, and incubated at room temperature in the dark for 15 min. Then, 400 µL of Binding Buffer was added and measured on the LSRII flow cytometer with 488 nm excitation wavelength (Becton Dickinson, San Jose, CA, USA). Cells incubated with 20 µM (HL-60 cells) or 100 µM (HL-60/DR cells) camptothecin (CAM) for 24 h were used as positive controls. The percentage of apoptotic cells presented in the results is the total amount of early (Q2) and late (Q3) apoptotic cells.

### 4.7. Gene Expression Analysis

Total RNA was extracted using the Universal RNA Purification Kit (EurX, Gdansk, Poland). The RNA concentration and purity were measured by BioTek Synergy HT Microplate Reader (BioTek Instruments, Winooski, VT, USA). The isolated RNA was stored at −20 °C until further steps. A total of 300 ng of RNA was used for one-step RT-qPCR reaction. Reverse transcription and real-time PCR reaction were performed using the SensiFAST™ Probe No-ROX One-Step Kit (Bioline, London, UK) on the CFX96 real-time system (Bio-Rad, Hercules, CA, USA). The relative expression of *NRF2*, *KEAP1*, *SOD2*, *HMOX2*, *GSS*, *CAS-3*, *CAS-8*, and *CAS-9* genes was evaluated using the TaqMan gene expression assay (ThermoFisher Scientific, Waltham, MA, USA). The analysis was performed in two biological replicates in two independent experiments. The *GAPDH* gene was utilized as a reference gene. Relative gene expression was calculated as a fold-change according to the control sample based on the double delta C_t_ method [61].

### 4.8. Western Blot

Following the treatment, cells were lysed in RIPA buffer (50 mM Tris HCl pH 8, 150 mM NaCl, 0.5% sodium deoxycholate, 1% Nonidet P-40, 0.1% SDS, 1 mM EDTA) with 1 mM phenylmethanesulfonyl fluoride (PMSF) and a protease and phosphatase inhibitor cocktail (Sigma-Aldrich, St. Louis, MO, USA). The protein concentration in cell lysates was determined using the Bradford method [62]. The 80 µg of proteins was separated on 10% SDS-PAGE and transferred to PVDF transfer membrane (ThermoFisher Scientific, Waltham, MA, USA). The membranes were blocked with 5% milk in 0.1% Tween-20/TBS (Tris-buffered saline) for 1 h at room temperature and they were incubated with rabbit monoclonal anti-NRF2 antibody (#12721, Cell Signaling Technology, Danvers, MA, USA), diluted 1:1000 in 5% milk in 0.1% TBST or anti-KEAP1 (#8047, Cell Signaling Technology, Danvers, MA, USA), diluted 1:1000 in 5% bovine serum albumin (BSA) in 0.1% TBST overnight at 4 °C. Goat anti-rabbit antibody conjugated with horseradish peroxidase (#7074, Cell Signaling Technology, USA) was used as a secondary antibody. For loading, the control detection of β-actin was performed with anti-β-actin antibody (sc-477778, Santa Cruz Biotechnology, Santa Cruz, CA, USA). The bands were visualized with Clarity Western ECL Substrate (Bio-Rad, Hercules, CA, USA) and Gel-Pro Analyzer (Microchem Laboratory, Round Rock, TX, USA). The band quantification was performed using ImageJ software v1.54g (National Institutes of Health, Bethesda, MD, USA) and the band intensities were normalized against β-actin.

### 4.9. Statistical Analysis

Experiments were conducted at least in duplicates. The data are presented as mean ± standard deviation (SD). The statistical analysis was conducted using the U Mann–Whitney test (samples with distributions departing from normality). The differences were considered to be statistically significant when the *p* value was <0.05.

## 5. Conclusions

In summary, we found that each tested NRF2 inhibitor—K67, RA, and ML-385—overcame doxorubicin resistance in HL-60/DR cells. We discovered that NRF2 inhibitors significantly improved the therapeutic efficacy of doxorubicin by increasing cytotoxicity, apoptosis, and intracellular ROS level. ML-385 appears to be the most effective NRF2 inhibitor in HL-60/DR cells due to the increased cytotoxicity of doxorubicin at lower concentrations than RA and K67. However, we identified K67, RA, and ML-385 as promising candidates for further investigations on the reversal of doxorubicin resistance in leukemia in vivo models and clinical trials.

## Data Availability

All data generated or analyzed during this study are included in this published article.
